# When cancer strikes (twice)

**DOI:** 10.7554/eLife.86758

**Published:** 2023-02-10

**Authors:** Nicole Swann

**Affiliations:** 1 Department of Human Physiology, University of Oregon Eugene United States

**Keywords:** sparks of change, being a scientist, cancer, equity diversity inclusion, motherhood, research culture

## Abstract

A young group leader reflects on academic culture and working while going through cancer treatments.

There it was, right in the pathology results. My worst fears come true. I was 37 years old, an assistant professor on the tenure track, a mother of two, and I had cancer. Again.

There was some good news. It was stage 3 colon cancer, which meant I could receive treatments that would give me good odds of survival. I was as lucky as someone with such a diagnosis could be, but I knew I had a long road ahead of me. I knew this because thirteen years prior, as a graduate student, I had been diagnosed with stage 3 breast cancer.

As the shock of the news sunk in, my mind started to scramble. I had just returned from maternity leave, I had a four-month-old baby and a four-year-old child… How would I make this work? Moments before, I had been struggling to imagine how, in the middle of a global pandemic, I would be able to balance my family with teaching and running a lab. It had all felt daunting, even with an incredibly supportive partner by my side. Now it seemed impossible.

I knew from experience that managing oncology treatments is a full-time job. I would soon be surrendering control over my calendar and helplessly watch it fill up with scans and appointments. I would be spending entire days in the infusion room to receive chemotherapy, before going home to pump in more drugs. There would be radiation treatment, which requires daily trips to the cancer center, as well as five surgeries and their associated recovery periods. Week after week, for over a year, I would be feeling miserable. Most of all, I knew that every time I picked up the phone or attended an appointment, I could receive news that would impact whether I might be around to see my kids grow up.

Taking a break in my career would make things easier, and perhaps I could make it happen. I was granted a tenure extension, I had access to paid sick days (including some I could borrow from myself) and I could take longer unpaid medical leave without losing my health insurance. But I had a lab to run, and we were working on grants which had inflexible deadlines. I just didn’t see how I could put my work on hold without letting down my students, my collaborators, and my future self. I already had several grants rejected citing my lack of publications after my first diagnosis and the birth of my eldest. Between my recent maternity leave and the COVID restrictions, I dreaded adding another career gap to my resume. I also had to consider how taking unpaid leave would impact my family financially.

I felt torn, but in the end, I decided to keep my research going. With the support of my department, I came up with a plan. I would take a break from teaching and service by taking a couple weeks of medical leave here and there; otherwise, I would attempt to work around my appointments with the help of the occasional sick day. In the end, this seemed the best way to keep my lab afloat while minimizing the impact on my career and my finances.

Some things were easier compared to when I was a graduate student. Now I had good insurance, a bit more control over my schedule, and more money. The health plan I had back then was ridiculously insufficient, and the fact that I was still on my parents’ insurance may have quite literally saved my life. I also couldn’t afford parking on campus at the time, and spots were rarely available for students anyway. I remember trudging between the lab and the cancer center on my old beat-up bike so I could receive my daily dose of radiation. It wasn’t a long ride, but exhaustion is a side-effect of the treatment and I sometimes felt like I was almost riding backwards. This time, I could drive to my appointments if I needed to. In truth, I have been lucky and very privileged. I have had access to resources and support that many other patients, if not most, do not receive.

And still, I struggled. I tried to do my best work while saving my best self for my family, but treatments drained every ounce of energy from me. Some days, my body simply had nothing left to give; at other times, my mind was filled with so much planning, fear and worry that I just didn’t have the mental clarity required for scientific thinking. Maybe I could answer an email, but there was no chance I could write a sensible grant.

Everyone around me was deeply sympathetic, yet the constant need for productivity managed to wriggle its way into our interactions and conversations. Once I was texting a friend, who is also an academic, as I was waiting in the emergency room with a post-surgical complication so painful that I could hardly keep my eyes open. In the middle of the pandemic, it took hours for someone to see me. She asked if I was at least able to get some work done while waiting, as if the situation might be tolerable if I was able to be productive. I don’t blame her; before I faced cancer, I might have asked the same. Now I see that academia is built on the assumption that trainees are young and healthy, that they are willing and able to be in the lab at all hours, to work for inadequate wages and to move away from their support networks. It is a system that excludes the most vulnerable, including those with chronic and serious illnesses.

I was young and by all accounts healthy when I received my diagnoses. I had no risk factors, no known exposures to carcinogens and no family history. According to extensive genetic testing, I don’t even carry any documented mutations associated with cancer. And yet this has happened to me. Twice. As researchers, we know that there are some questions we just can’t answer; there is always a mysterious data point that doesn’t make sense, that we can’t explain. It’s different when, suddenly, *you are* that data point. I often felt compelled to read the relevant scientific literature, which led me to obsess over every test result, every word in my medical reports. Even when I knew the findings were unlikely to be clinically significant, at least at the individual level, I still found myself scrutinizing them. It was stressful and ultimately pointless.

I finished my treatments several months ago. My scans all look good – “no evidence of disease”. My doctors and I are optimistic that it will stay that way. Looking back, I think that my decision to keep working was the right one for me given my circumstances. Now that I have the mental space to think about my research, I am grateful that my projects stayed afloat so I don’t have to start from square one. Yet going through it all was tremendously difficult. This is not to say that my university, my department, or my colleagues fell short – they did everything they could to accommodate me within a research culture that needs revising. I don’t regret my choice, but I wish I had had a different one; I wish academia had ways for us to take the breaks we all need in life without harming our students, our labs and our careers.

Odds are good that I will stay in remission, but I don’t think anyone can face two stage 3 cancers and not think about mortality. Academia often entails “playing the long game". We accept struggle now because we hope that the next career stage will be less stressful, more rewarding. I don’t tolerate much of that anymore. I won’t allow myself to sacrifice my present for a future I may not have, a future none of us can take as a given. I work hard at my job, and I enjoy it. I just won’t put my life aside for it. Perhaps this means that I won’t perform as well as I had once hoped in my career, but I cannot let this fear cause me to miss out on life. If I fail – fail to publish where I’d wanted, fail to get ‘the grant’, even fail to get tenure – at least it means that I am still here. Here to receive such disappointing news, but also here to watch my kids get older, here for another anniversary, another birthday, another milestone. Here to hope for more time. More time for science… but above all, more time for living.

## Share your experiences

This article is a Sparks of Change column, where people around the world share moments that illustrate how research culture is or should be changing. Have an interesting story to tell? See what we’re looking for and the best ways to get in touch here.

